# Efficacy and safety of nebulized inhalation vs. intramuscular delivery of interferon α1b injection for paediatric patients with viral respiratory diseases: a systematic review and meta-analysis

**DOI:** 10.3389/fped.2025.1654973

**Published:** 2025-10-23

**Authors:** Lixing Yang, Lu Cao, Yaning Zhu, Ying Zhao, Peng Zhang

**Affiliations:** Department of Pharmacy, Shaanxi Provincial People’s Hospital, Xi’an, Shaanxi, China

**Keywords:** interferon α1b, nebulized inhalation therapy, paediatric viral respiratory diseases, efficacy, safety

## Abstract

**Objectives:**

To systematically evaluate the efficacy and safety of nebulized inhalation vs. intramuscular delivery of interferon α1b (IFN α1b) for paediatric patients with viral respiratory diseases.

**Methods:**

A comprehensive search of databases including PubMed, Web of Science, Cochrane, Embase, China National Knowledge Infrastructure (CNKI), and China Biology Medicine disc (Sinomed) was conducted to identify relevant literature on the use of interferon α1b in children. The search timeframe spanned from database inception to April 2025.

**Results:**

A total of 16 studies involving 2002 patients were included. The meta-analysis revealed that the overall efficacy rate in the nebulized inhalation group (94.85%) was significantly greater than that in the intramuscular injection group (82.39%) (*P* < 0.00001). Consistent results were observed in the herpangina and bronchiolitis subgroup analyses (*P* < 0.0001). With respect to drug safety, the meta-analysis results revealed that the incidence rate of adverse reactions in the nebulized inhalation group (1.58%) was significantly lower than that in the intramuscular injection group (4.60%) (*P* = 0.003). The studies had no significant publication bias, and sensitivity analysis suggested that the results were reliable.

**Conclusion:**

Compared with intramuscular injection, nebulized inhalation significantly increased the efficacy and safety of IFN α1b in treating paediatric patients with viral respiratory diseases. For children with both herpangina and bronchiolitis, nebulized inhalation was more effective; however, no significant difference was found in the incidence of adverse reactions. In the future, multicentre, large-scale randomized controlled trials should be conducted to further validate these conclusions.

## 1 Introduction

Children's immune systems are not yet fully developed; in particular, they have lower levels of specific immunity, cellular immunity, and interferon (IFN), making viral diseases common among children. Common respiratory viral infections in children include the common cold, influenza, herpangina, bronchitis, and pneumonia. Pathogenic viruses that are frequently diagnosed include respiratory syncytial virus (RSV), human rhinovirus (HRV), parainfluenza virus (PIV), influenza virus (IV), adenovirus (ADV), human metapneumovirus (HMPV), cytomegalovirus (CMV), and coronavirus (CoV) (1, 2). In pneumonia cases, viruses account for 55% of the primary pathogens (3). Currently, specific therapeutic agents are available for infections caused by influenza A and B viruses, cytomegalovirus, herpes simplex virus, and varicella-zoster virus, but effective treatments are lacking for other viral infections.

Recombinant human interferon α1b (rhIFNα1b) injection is a broad-spectrum antiviral drug, and its indications listed in the prescribing information include the treatment of paediatric RSV pneumonia (4). Additionally, IFN-α injections are widely used clinically for treatment of diseases caused by other respiratory viruses, such as IV, ADV, and CoV, and have demonstrated good efficacy (5–7). Other broad-spectrum antiviral drugs, such as the synthetic nucleoside Ribavirin, are associated with numerous adverse effects, including haemolytic anaemia, decreased haemoglobin levels, anaemia, liver function impairment, and fatigue (8, 9), and are therefore not routinely recommended for the treatment of viral respiratory infections in children.

According to the prescribing information, recombinant human IFN α1b injection is administered via direct intramuscular (im), subcutaneous, or lesion injection. However, in clinical practice, nebulized inhalation (inh) of IFN α1b is frequently used empirically for treating paediatric patients with respiratory viral infections, which means that the indications, patient groups, and administration routes are not within the scope approved by the drug regulatory authorities and that it is considered “off-label use”. Compared with intramuscular injection, nebulized inhalation has distinct advantages (10): (1) aerosolized particle deposition directly in the airways and lungs with increased targeting specificity; (2) rapid distribution, higher concentration, and prolonged retention in lung tissues; (3) improved safety profile, unlike intramuscular injection, which may induce initial flu-like symptoms and, with prolonged use, risks bone marrow suppression or psychiatric disturbances, with nebulized inhalation, in contrast, being generally well tolerated with minimal adverse effects; and (4) ease of administration and higher compliance in paediatric patients, facilitating clinical adoption. Literature reviews indicate that as early as the 1980s, interferon and its inducers, when administered via nebulizer inhalation, demonstrated significant efficacy in treating patients with viral pneumonia ^(^11). In recent years, guidelines and expert consensus have proposed that the clinical use of IFN-α through spray or nebulized inhalation for local treatment can aid in viral clearance, shorten the disease course, alleviate symptoms, and result in a favourable safety profile (12). Nevertheless, systematic studies comparing the efficacy and safety of nebulized inhalation vs. intramuscular injection are lacking.

In our study, a meta-analytic approach was employed to systematically compare the differences in overall efficacy rate and adverse reaction incidence rate between nebulized inhalation and intramuscular injection administration routes. This comparative evaluation aims to assess the clinical efficacy of nebulized interferon therapy for paediatric patients with viral respiratory diseases, with the goal of providing high-quality, evidence-based medical support for clinical use.

## 2 Materials and methods

### 2.1 Literature search strategy

A comprehensive search of English databases, including PubMed, Web of Science, Cochrane, Wiley, Elsevier, and Embase, as well as Chinese databases, such as China National Knowledge Infrastructure (CNKI), Wanfang Data, VIP, China Biology Medicine disc (Sinomed), and Chinese Medical Journal Full-text Database, was conducted to identify relevant literature on the use of interferon α1b in children. The search strategy employed a combination of Medical Subject Headings (MeSH) terms and free-text words, tailored to the specificities of each database. The search timeframe spanned from the inception of each database to April 2025. Keywords included “interferon”, “infant”, “child”, “neonate”, and “newborn”. Taking PubMed as an example, the search strategy was as follows:

#1 Interferon
#2 Infant OR child OR neonate OR newborn
#3 #1 AND #2

### 2.2 Inclusion criteria

1. Participants: Patients aged ≤14 years who were admitted to the paediatric department, were diagnosed with viral respiratory infections, and were treated with interferon α1b for antiviral therapy, regardless of sex.
2. Interventions/Comparisons: Different administration methods of interferon α1b were compared, with the intervention group receiving nebulized inhalation and the control group receiving intramuscular injection. There were no restrictions on the dosage administered.
3. Outcomes: The primary effectiveness outcome was the overall efficacy rate, which was defined as the percentage of effective cases (including markedly effective cases and effective cases) among the total number of evaluated cases. The criteria for efficacy assessment vary by disease population (Table 1).
  The safety outcome was the adverse reaction incidence rate, which was defined as the percentage of individuals with adverse reactions among the total exposed population.
4. Study Types: Published randomized controlled trials (RCTs), as well as prospective or retrospective studies, were included.

**Table 1:** Efficacy evaluation criteria for included diseases.

Disease	Markedly effective	Effective	Ineffective
Herpangina	Within 2–3 days of treatment, the body temperature returns to normal, oral herpes shrinks significantly, sore throat and salivation disappear, no ulcers form, and the appetite and mental state improve.	Within 4–5 days of treatment, the body temperature basically returns to normal, oral herpes shrinks, sore throat and salivation disappear, no ulcers form, and the appetite and mental state improve.	After 5 days of treatment, the fever does not subside or the body temperature rises; sore throat and salivation persist or worsen; oral herpes does not shrink or increases in size; and ulcers form.
Bronchiolitis	Within 5–7 days of treatment, clinical symptoms such as wheezing, rales, fever, and cough completely disappear; the heart rate is <120 beats per minute; the respiratory rate is <40 breaths per minute; and chest x-ray shows complete absorption of pulmonary inflammation.	Within 5–7 days of treatment, clinical symptoms including wheezing, rales, fever, and cough are relieved or improved to some extent; chest x-ray shows partial absorption of pulmonary inflammation.	Within 5–7 days of treatment, clinical symptoms like wheezing, rales, fever, and cough show no significant improvement or even worsen; chest x-ray shows aggravation of pulmonary inflammation.
Viral pneumonia	The patient's clinical symptoms such as elevated body temperature, dry cough, headache, and sore throat all disappear completely; chest x-ray shows the lungs return to normal; and moist rales are no longer heard on lung auscultation.	The patient's clinical symptoms including elevated body temperature, dry cough, headache, and sore throat are improved to some extent; chest x-ray shows the lungs basically return to normal; and moist rales are alleviated on lung auscultation.	The patient's clinical symptoms like elevated body temperature, dry cough, headache, and sore throat show no change or even worsen; chest x-ray shows abnormal lung conditions; and moist rales persist or become severe on lung auscultation.
Viral respiratory infections	Clinical symptoms and signs such as fever and cough completely disappear or are significantly relieved.	Clinical symptoms and signs including fever are improved to some extent.	Clinical symptoms and signs such as fever remain unchanged or worsen.

### 2.3 Exclusion criteria

1. Animal studies;
2. *In vitro* or cell culture studies;
3. Studies with unclear or unavailable raw data;
4. Studies involving participants who were not diagnosed with viral respiratory infections;
5. Interventions that did not align with the inclusion criteria;
6. Duplicate or repetitive studies;
7. Studies that utilized interferons other than the specified interferon α1b;
8. Studies that did not report predefined outcome measures or had ambiguous definitions of outcomes;
9. Literature limited to case reports without comparative or experimental data.

### 2.4 Data screening and extraction

In accordance with the predefined inclusion and exclusion criteria, two independent researchers screened the identified literature. Discrepancies in screening decisions were resolved through discussion. If a consensus could not be reached, a third researcher was consulted to make the final decision. Data extraction was performed on the included studies, focusing on the following aspects:

1. General study information: title, first author, publication date, and study design.
2. Clinical characteristics of the study population: disease type, participant age, and sample size.
3. Intervention and control measures: administration route, dosage, frequency, and duration of treatment.
4. Study outcomes: effectiveness and safety outcomes as defined by the respective indicators.
5. Quality assessment of the included studies.

### 2.5 Quality assessment of the included studies

The methodology quality of randomized controlled trials (RCTs) was assessed using the modified Jadad scale, which includes scoring of randomization, concealment of allocation, double blinding and withdrawals and dropouts. Studies scoring 1–3 points were classified as low quality, while those scoring 4–7 points were considered high quality.

The quality of the retrospective studies was evaluated using the Newcastle‒Ottawa Scale (NOS). which assesses the representativeness of the study population, the comparability of study groups, the adequacy of follow-up, and the completeness of outcome reporting. Higher scores indicate a lower risk of bias. Studies scoring between 5 and 10 points, which are considered to have minimal bias, were included in the meta-analysis.

### 2.6 Statistical analysis

Statistical analysis was performed using Review Manager (RevMan) version 5.4. For dichotomous outcomes, the odds ratio (OR) and its 95% confidence interval (CI) were calculated. Heterogeneity among the included studies was assessed using the Cochran *Q*-test: if *P* was >0.1 and I^2^ was ≤50%, indicating no significant heterogeneity, a fixed-effects model was applied; otherwise, a random-effects model was used. Publication bias for outcomes was assessed by inspection of the funnel plot symmetry. Sensitivity analyses were conducted to verify the robustness of the results using Stata 15.0 software.

## 3 Results

### 3.1 Literature search results and methodological quality assessment

**Figure 1:**
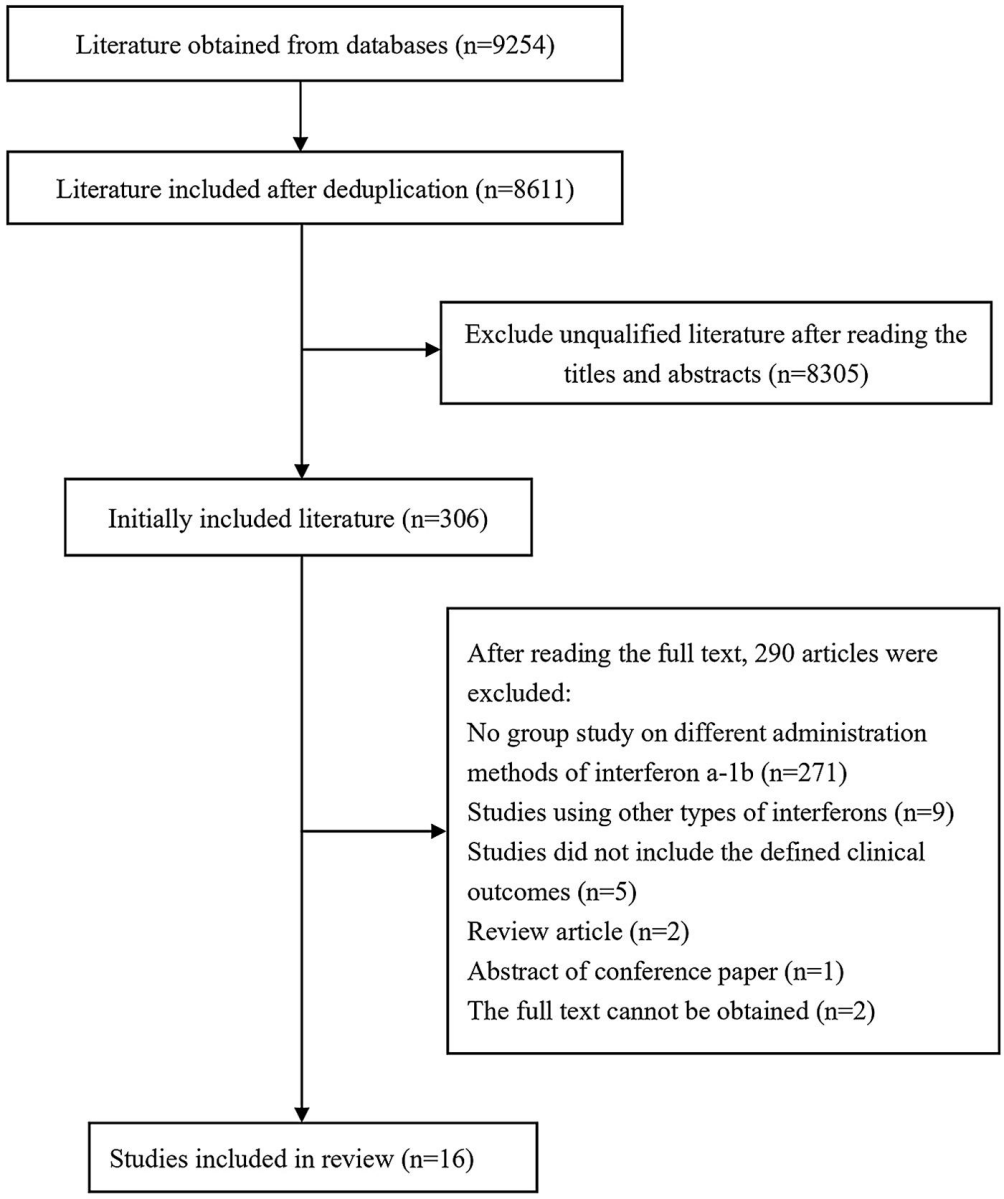
Literature screening flowchart.

On the basis of the predefined search strategy, a total of 9,254 articles were retrieved from the Chinese and English databases, including 4,514 English articles and 4,740 Chinese articles. The distribution across databases was as follows: PubMed (2,003 articles), Web of Science (1,144 articles), Cochrane (1,130 articles), Wiley (215 articles), Elsevier (22 articles), CNKI (496 articles), Wanfang (428 articles), VIP (1,801 articles), Chinese Medical Journal Full-text Database (12 articles), and Sinomed (2,003 articles). After duplicates were removed, 8,611 articles remained. Following a review of titles and abstracts, 306 articles were selected for further evaluation, and ultimately, 16 articles were included for systematic review and meta-analysis (Figure 1).

Among the 16 included studies, 15 were prospective randomized controlled trials (RCTs). Methodological quality assessment using the modified Jadad scale revealed that 3 prospective RCTs were of high quality (scoring 4–7 points), whereas 12 were of lower quality (scoring 1–3 points). The remaining retrospective cohort study scored 7 points on the Newcastle‒Ottawa Scale (NOS). The detailed scoring results are presented in Table 1.

### 3.2 Characteristics of the included studies

The final analysis included a total of 2002 patients, with 923 in the control group (intramuscular injection) and 1,079 in the intervention group (nebulized inhalation). In the design of each study, both the intervention and control groups were balanced with respect to baseline characteristics and disease types, ensuring comparability between the groups. The disease types among the study participants included herpangina, bronchiolitis, viral pneumonia and respiratory infections. The basic characteristics of the included studies are summarized in Table 2.

**Table 2:** Characteristics of included studies.

Author, year	Study design	Age included	Study population	Control (sample size)	Intervention (sample size)	Outcome measures	Modified Jadad scale/NOS score
Liu, 2019 (13)	Prospective RCTs	6 months–3 years	Herpangina	2 or 4 μg/kg, im, qd, 5 d (80)	2 or 4 μg/kg, inh, bid, 5 d (80)	①②	2
Li, 2020 (14)	Prospective RCTs	2 months–4 years	Viral pneumonia	1 or 2 μg/kg, im, qd, 5–7 d (56)	1–2 or 2–4 μg/kg, inh, bid, 5–7 d (56)	①②	3
Ma, 2019 (15)	Prospective RCTs	3 months–6 years	Bronchiolitis	1 μg/kg, im, qd, 7 d (45)	2 ug/kg, inh, bid, 7 d (45)	①②	3
Yan, 2020 (16)	Prospective RCTs	6 months–6 years	Bronchiolitis	1 μg/kg, im, qd, 7 d (50)	2 μg/kg, inh, bid, 7 d (50)	①	3
He, 2018 (17)	Prospective RCTs	1–8 years	Herpangina	10 or 20 μg, im, qd, 3–5 d (28)	10 or 20 μg, inh, qd, 3–5 d (28)	①②	4
Ding, 2020 (18)	Prospective RCTs	1–24 months	Bronchiolitis	1 μg/kg, im, qd, 5–7 d (32)	1 μg/kg, inh, bid, 5–7 d (32)	①②	2
Zhang, 2018 (19)	Prospective RCTs	1–13 years	Herpangina	im, dose not mentioned (40)	inh, dose not mentioned (40)	①②	2
Ji, 2021 (20)	Prospective RCTs	1–10 years	Herpangina	10 or 20μg, im, qd, 5 d (60)	10 or 20μg, inh, qd, 5 d (60)	①	2
Han, 2022 (21)	Retrospective cohort study	6 months–3 years	Bronchiolitis	1 μg/kg, im, bid, 7 d (43)	1 μg/kg, inh, bid, 7 d (43)	①②	7
Ou, 2015 (22)	Prospective RCTs	1–14 years	Viral respiratory infections	0.5–1μg/kg, im, qd, 3–5 d (80)	0.5–1μg/kg, inh, bid, 3–5 d (80)	①②	2
Xu, 2016 (23)	Prospective RCTs	0–24 months	Bronchiolitis	1 μg/kg, im, bid, 7d (35)	1 μg/kg, inh, bid, 7 d (42)	①	2
Wang, 2021 (24)	Prospective RCTs	2.5 months–2.5 years	Bronchiolitis	1 μg/kg, im, qd, 7 d (55)	2 μg/kg, inh, bid, 7 d (55)	①	2
Si, 2019 (25)	Prospective RCTs	1–3 years	Bronchiolitis	1 μg/kg, im, bid, 7 d (49)	1 μg/kg, inh, bid, 7 d (49)	①	3
Wang, 2018 (26)	Prospective RCTs	8 months–7.3 years	Herpangina	1 μg/kg, im, qd, 5–7 d (42)	2–4 μg/kg, inh, qd, 5–7 d (41)	②	2
Chen, 2020 (27)	Multicenter prospective RCTs	0–12 months	Bronchiolitis	10 μg, im, qd, 7 d (150)	1 or 2 μg/kg, inh, bid, 7 d (300)	②	5
Huang, 2016 (28)	Prospective RCTs	0.5–13 years	Bronchiolitis	1 μg/kg, im, qd, 5–7 d (78)	1 μg/kg, inh, bid, 5–7 d (78)	②	4

### 3.3 Meta-analysis results

#### 3.3.1 The overall efficacy rate

Thirteen studies (13–25) compared the overall efficacy rate of nebulized inhalation vs. intramuscular delivery of IFN α1b injection in the treatment of paediatric patients with viral respiratory diseases.

**Figure 2:**
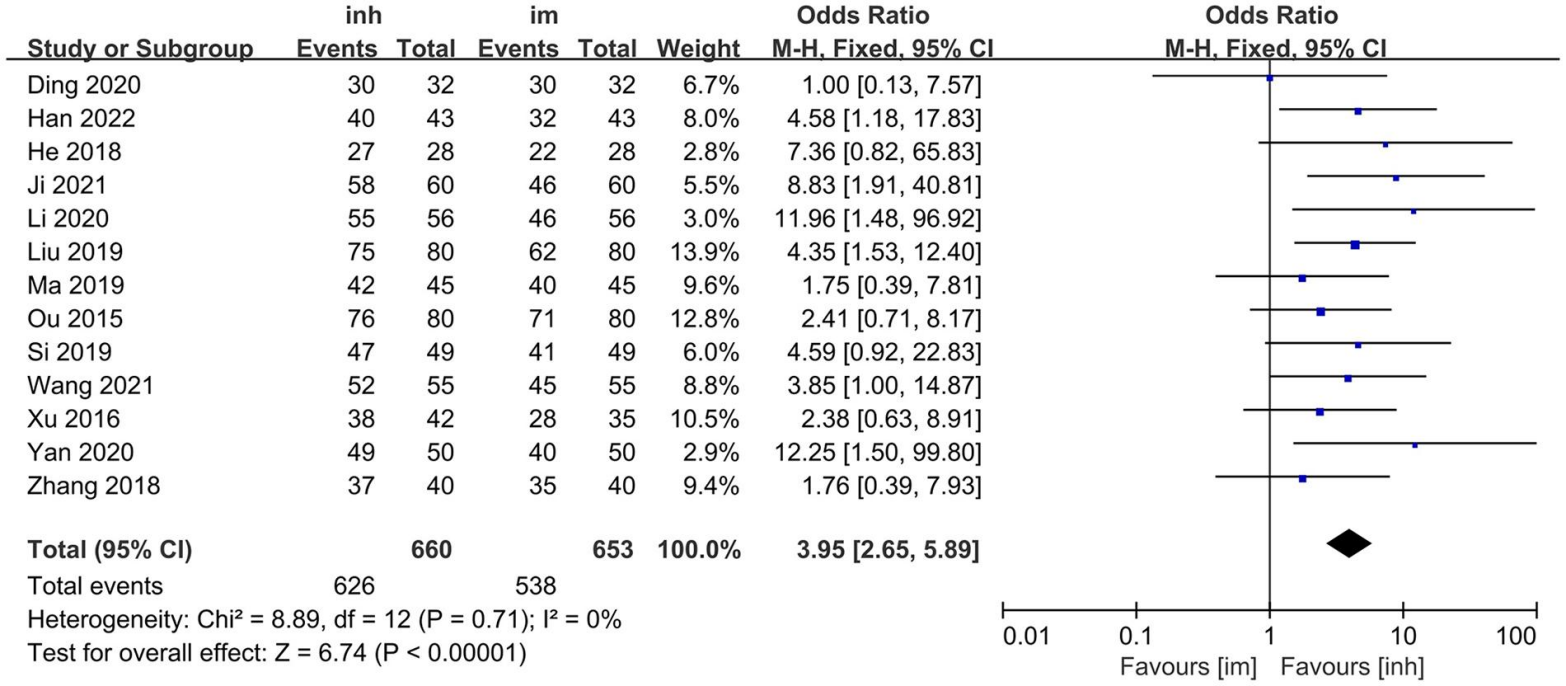
Overall efficacy rate in inh and im groups.

No significant heterogeneity was observed among the included studies (*P* = 0.71; I^2^ = 0%), and thus, a fixed-effects model was employed for the analysis. The meta-analysis results demonstrated that the overall efficacy rate in the nebulized inhalation group (94.85%) was significantly greater than that in the intramuscular injection group (82.39%). The difference between the two groups was statistically significant [OR: 3.95; 95% CI: 2.65–5.89; *P* < 0.00001] (Figure 2).

#### 3.3.2 Subgroup analysis of overall efficacy rate

Among the 13 included studies, 7 focused on children with herpangina and 4 on children with bronchiolitis, and the remaining 2 did not specify the disease type. Subgroup analyses were performed for herpangina and bronchiolitis.

##### 3.3.2.1 Herpangina subgroup

**Figure 3:**
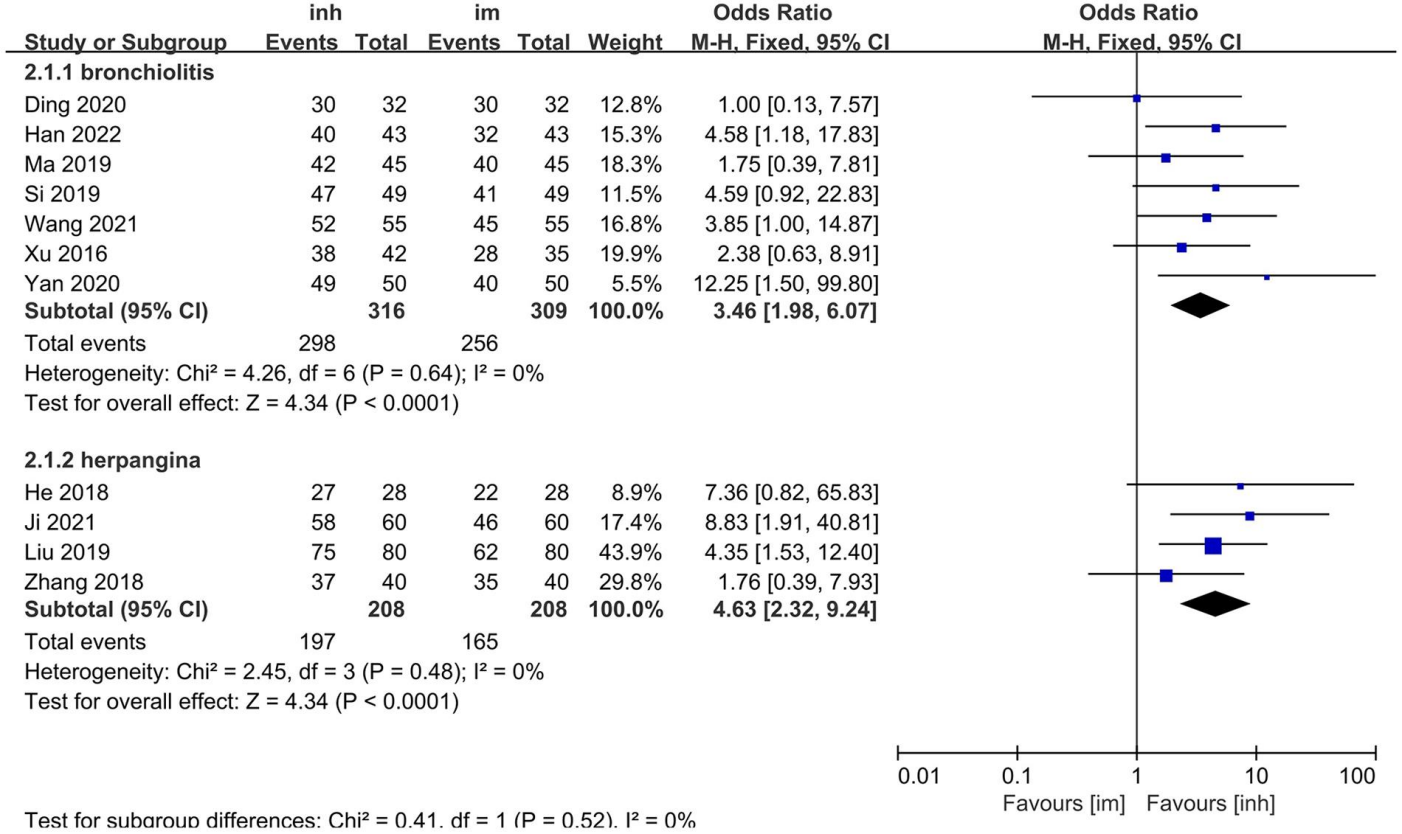
Subgroup analysis of overall efficacy rate in inh and im groups.

No significant heterogeneity was observed among the studies (*P* = 0.64; I^2^ = 0%), so a fixed-effects model was applied. The meta-analysis demonstrated that the nebulization group had a significantly higher overall efficacy rate (94.30%) than the intramuscular injection group did (82.85%). The difference was statistically significant [OR: 3.46; 95% CI: 1.98–6.07; *P* < 0.0001] (Figure 3).

##### 3.3.2.2 Bronchiolitis subgroup

Similarly, no significant heterogeneity was detected (*P* = 0.48; I^2^ = 0%), and a fixed-effects model was used. The overall efficacy rate (94.71%) was significantly greater in the nebulized inhalation group than in the intramuscular injection group (79.32%) [OR: 4.63, 95% CI: 2.32–9.24; *P* < 0.0001] (Figure 3).

#### 3.3.3 Adverse reaction incidence rate

**Figure 4:**
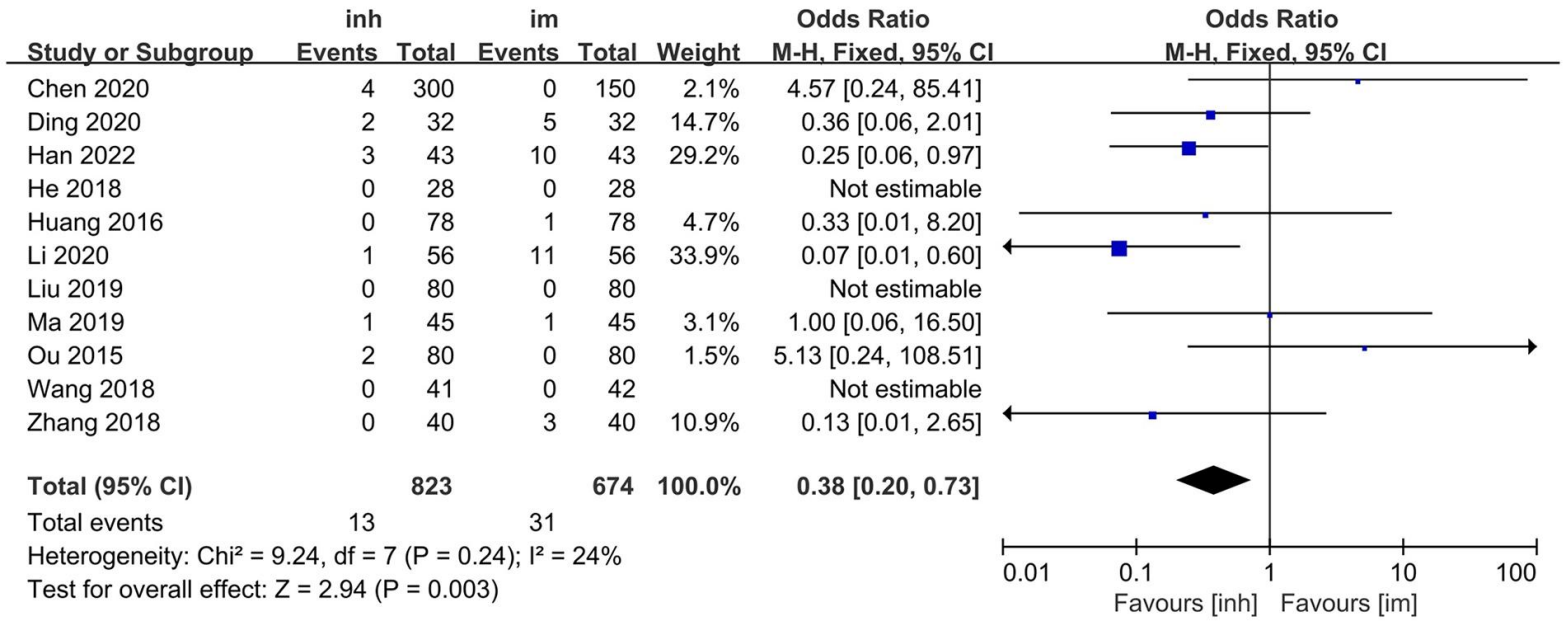
Adverse reaction incidence rate in inh and im groups.

Eleven studies (13–15, 17–19, 21–22, 26–28) compared the adverse reaction incidence rate between nebulized inhalation and intramuscular injection delivery of IFN α1b in the treatment of paediatric patients with viral respiratory diseases. No significant heterogeneity was observed among the included studies (*P* = 0.24; I^2^ = 24%), and thus, a fixed-effects model was utilized for the analysis. The results of the meta-analysis revealed that the incidence of adverse reactions in the nebulized inhalation group (1.58%) was significantly lower than that in the intramuscular injection group (4.60%). The difference between the two groups was statistically significant [OR: 0.38; 95% CI: 0.20–0.73; *P* = 0.003] (Figure 4).

### 3.4 Publication bias

**Figure 5:**
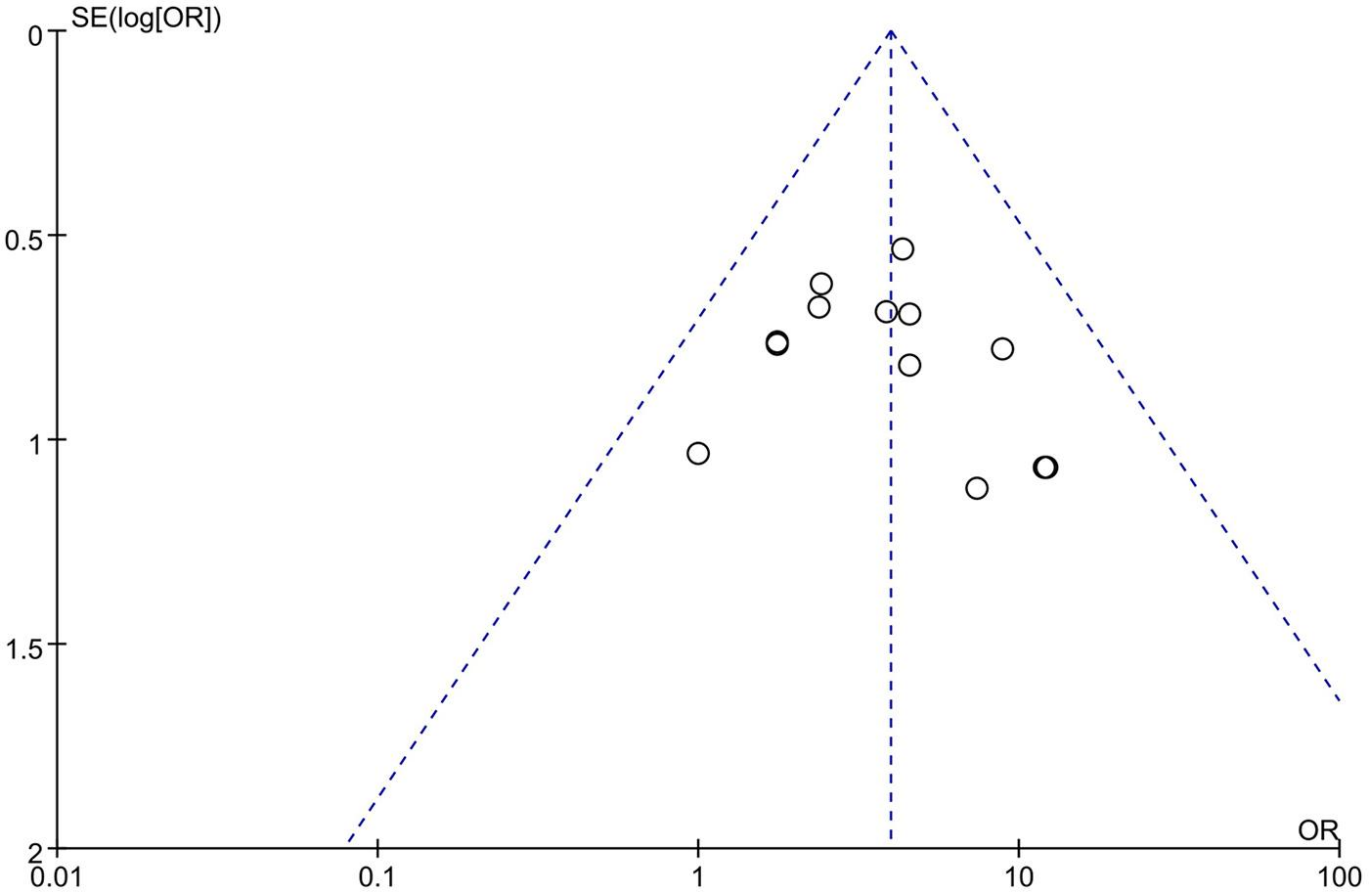
Publication bias for the effectiveness outcome measures.

**Figure 6:**
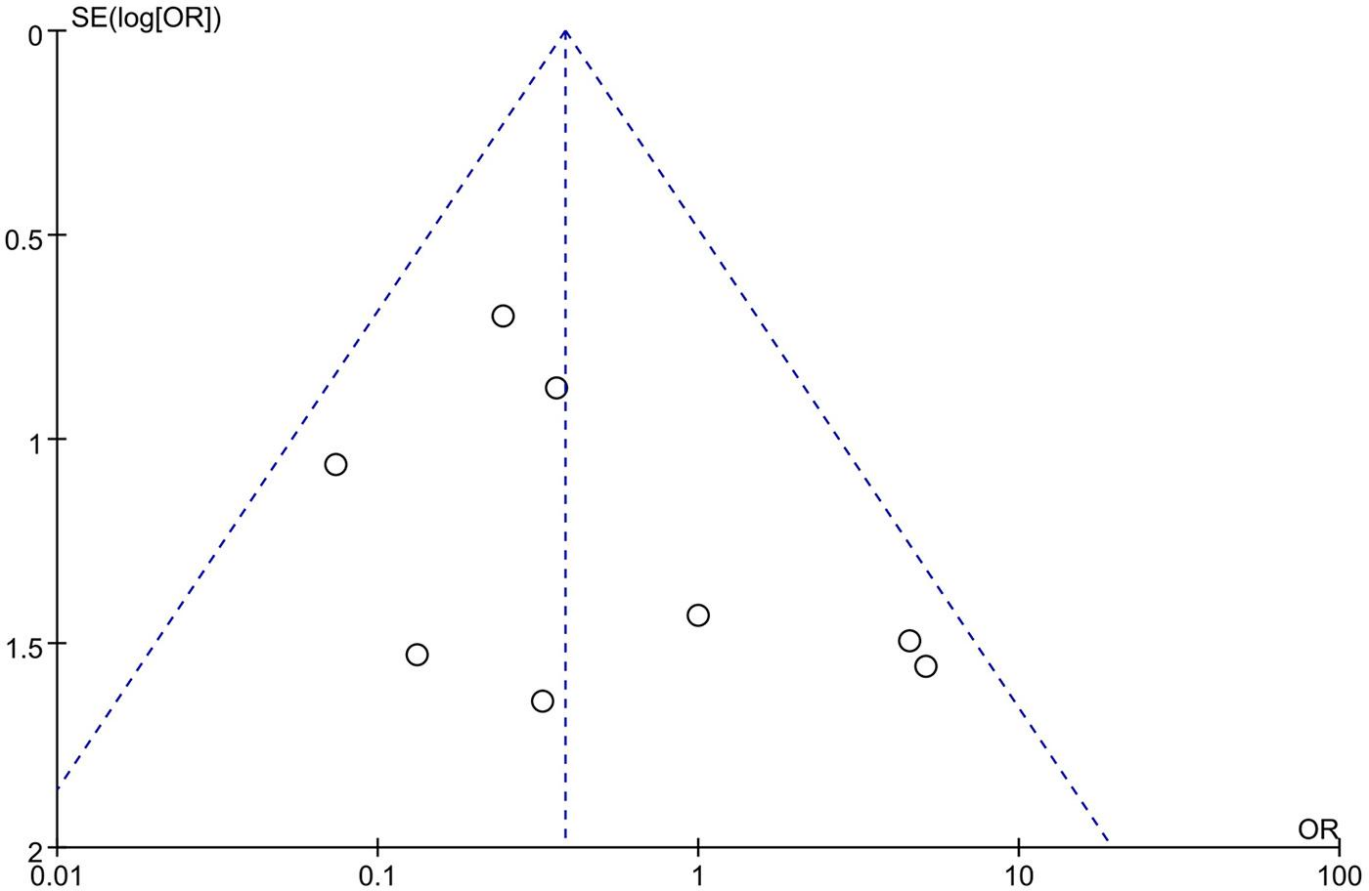
Publication bias for the safety outcome measures.

The funnel plots for the effectiveness and safety outcome measures demonstrated no significant asymmetry, suggesting a low likelihood of publication bias in this meta-analysis (Figures 5, 6).

### 3.5 Sensitivity analysis

**Figure 7:**
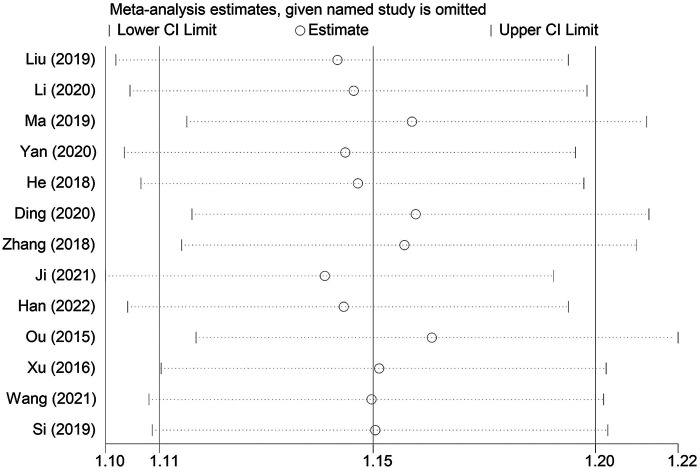
Sensitivity analysis of the effectiveness studies.

**Figure 8:**
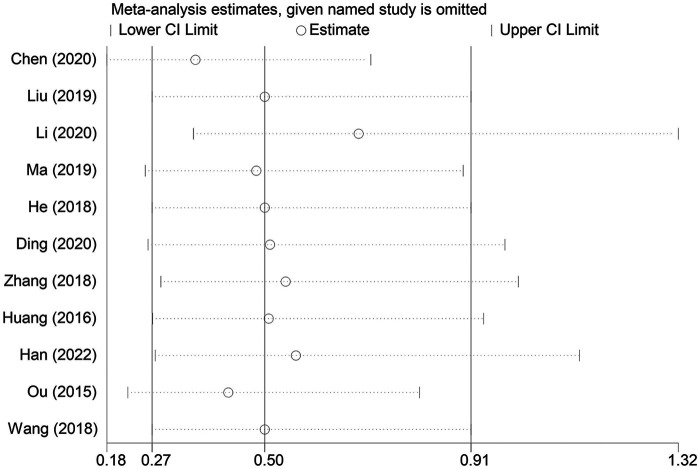
Sensitivity analysis of the safety studies.

A sensitivity analysis was conducted to examine the influence of individual studies on the overall pooled effect. If the results remained unchanged after sensitivity analysis, it would suggest that the meta-analysis results were robust. If the sensitivity analysis revealed significant changes, it would indicate the presence of potential factors related to the intervention that could affect the credibility of the results. Sensitivity analysis was performed on the overall efficacy rate and the adverse reaction incidence rate (Figures 7, 8), and the results revealed no substantial changes in the pooled effect estimate, suggesting that the meta-analysis results were reliable.

## 4 Discussion

Interferons (IFNs) are soluble glycoproteins that have multiple biological functions and are synthesized primarily by monocytes and lymphocytes. These cytokines are classified into three categories on the basis of their receptor specificity: type I (encompassing IFN-α, IFN-*β*, IFN-κ, IFN-δ, IFN-ε, IFN-τ, IFN-ω, and IFN-ζ); type II (IFN-γ); and type III (IFN-λ1, IFN-λ2, and IFN-λ3) (29). Among these classes, type I interferons mediate their biological effects through binding to a heterodimeric cell surface receptor complex known as IFN-α/β receptor (IFNAR) (30). In contrast to the restricted expression profiles of type II and type III interferon receptors, IFNAR subunits are ubiquitously expressed across nearly all immune cell lineages and epithelial tissues (31). This pervasive receptor distribution underscores the broad-spectrum immunomodulatory potential of type I IFNs, enabling them to coordinate pansystemic immune responses and thereby facilitate the rapid mobilization of systemic immune activation.

Innate immunity, also referred to as natural immunity, not only serves as the primary defence barrier against microbial pathogens but also profoundly influences the induction of adaptive immune responses (32). During the initial phase of acute infection, the production of type I interferons (IFN-I) and other inflammatory cytokines constitutes a pivotal event that critically determines the kinetics of viral replication and dissemination (33). Furthermore, IFN-I secretion plays a crucial role in modulating immune homeostasis, demonstrating significant immunoregulatory properties. In clinical scenarios where endogenous interferon production is insufficient to effectively eliminate viral pathogens, the administration of exogenous interferon preparations represents a viable therapeutic strategy to increase antiviral defences and immune competence.

Currently, the most widely used type I interferon in clinical practice in China is IFN-α, which primarily includes IFN α1b and IFN α2b. This systematic analysis focuses on IFN α1b as the investigational agent. Recombinant human IFN α1b is a broad-spectrum therapeutic agent with antiviral, antitumour, and immunomodulatory properties. In accordance with its prescribing information, the approved indications include the treatment of certain malignant neoplasms (such as chronic myeloid leukaemia, hairy cell leukaemia, melanoma, and lymphoma) as well as viral diseases (including chronic hepatitis B and C, herpes zoster, condyloma acuminatum, and epidemic haemorrhagic fever). In paediatric practice, IFN α1b has demonstrated confirmed therapeutic efficacy and broad clinical potential in the treatment of viral pneumonia, viral hepatitis, bronchiolitis, herpangina, hand-foot-and-mouth disease, and certain malignancies. In clinical practice in China, IFN α1b injection is used for nebulization because of the lack of a dedicated inhalation agent. Encouragingly, regulatory-compliant, specifically formulated nebulizable interferon solutions have now entered phase III clinical trials (34), and the first human IFN α1b inhalation solution, GB05, was developed in compliance with FDA guidelines (35).

Many studies have confirmed that the inhalation of IFN α1b is safe and effective for treating paediatric patients with viral respiratory diseases (compared with normal saline control) (36, 37). According to the “Guidelines for the rational use of antiviral drugs in children with respiratory viral infectious diseases” (38), IFN-α can be used for acute upper and lower respiratory tract infections caused by viruses in children. Inhalation of IFN-α can be used for acute lower respiratory tract viral infections in children. In medical institutions without inhalation equipment, children with bronchiolitis or viral pneumonia can be treated with IFN-α by intramuscular injection as appropriate. The “Expert consensus on inhalation therapy for common respiratory diseases in children” (39) and the “Guidelines for standardized management of children's inhalation centers” (40) indicate that IFN-α is a commonly used antiviral drug and has a history of clinical application. The “Chinese pediatric guideline for the diagnosis, treatment, and prevention of respiratory syncytial virus infection” (41) proposes that recombinant human IFN-α inhalation is safe and effective for RSV-related lower respiratory tract infections and recommends its use. The “Guidelines for the management of community-acquired pneumonia in children (2024 revision)” (12) state that interferon has a broad-spectrum antiviral effect and can be used to treat viral pneumonia. The “Expert consensus on the rational application of interferon alpha in pediatrics” (42) mentions that subcutaneous or intramuscular injection of IFN-α drugs can be distributed throughout the body and is used in clinical practice to treat various viral infections and haematological diseases in children. Inhalation of IFN-α drugs leads to distribution mainly in the respiratory tract and is used to treat various respiratory viral infections. The “Diagnosis, treatment and prevention of severe acute respiratory syndrome coronavirus 2 infection in children: experts’ consensus statement (Fourth Edition)” (43) suggests that IFN-α inhalation can be used for children with pneumonia and other lower respiratory tract infections. Beyond the recommendations in guidelines and consensuses, preclinical and clinical data have been published on the advantages, but there is still a lack of systematic reviews to determine whether it has significant advantages over intramuscular injection.

In our study, the overall efficacy rate of nebulized inhalation of IFN α1b in the treatment of paediatric patients with viral respiratory diseases was significantly greater than that of intramuscular injection. Subgroup analyses revealed that nebulized inhalation was more effective for both herpangina and bronchiolitis patients. In terms of the incidence of adverse reactions, nebulized inhalation resulted in a significantly lower incidence than intramuscular injection did. The types of adverse reactions during intramuscular injection were more diverse and included nausea, vomiting, fever, chills, local redness at the injection site, rash, headache, listlessness, and granulocytopenia. The adverse reactions to nebulized inhalation of IFN α1b mainly included rash, listlessness, fever, nausea, and vomiting. However, in the subgroup analysis, there was no significant difference between the herpangina and bronchiolitis groups (Supplementary Figure S1), which might be due to the reduced sample size in the subgroups, leading to a decrease in statistical power.

This study has several limitations: (1) the quality assessment scores of the included studies were not high, largely because of the inability to implement blinding in the administration methods; (2) most of the included studies were single-centre studies, which might have a certain influence on the universality of the final conclusion; (3) owing to the numerous viral respiratory diseases in children and the large number of outcome indicators in various studies that are difficult to unify, only the overall efficacy rate is adopted as the effectiveness indicator; and (4) the heterogeneity of efficacy criteria in different diseases might influence the generalizability of the efficacy findings and limit the comparability and interpretability of the pooled efficacy estimate, despite the statistical homogeneity (low I^2^).

## 5 Conclusion

In summary, the current evidence demonstrates that nebulized inhalation of recombinant human interferon α1b (injection form) is safer and more effective than intramuscular injection in the treatment of paediatric patients with viral respiratory diseases, providing substantial support for this off-label clinical application. However, we strongly recommend that future multicentre, large-scale randomized controlled trials be conducted to further validate the comparative efficacy and safety of these two administration routes.

Although our findings suggest favourable safety outcomes with nebulized administration, strict adherence to standardized nebulization protocols and relevant clinical guidelines remains imperative. This includes proper administration techniques, rigorous monitoring for potential adverse reactions, and the implementation of appropriate preventive measures against treatment-related complications. In the future, we anticipate the development and approval of dedicated nebulized formulations.

## Data Availability

The original contributions presented in the study are included in the article/Supplementary Material, further inquiries can be directed to the corresponding author.

## Supplementary material

Supplementary Figure S1 Subgroup analysis of adverse reaction incidence rate in inh and im groups.
