# Supplementary figures and images for: Efficacy and safety of nebulized inhalation vs. intramuscular delivery of interferon α1b injection for paediatric patients with viral respiratory diseases: a systematic review and meta-analysis
Source: Front Pediatr. 2025 Oct 23;13:1654973. doi: 10.3389/fped.2025.1654973 (PMC12588897; doi:10.3389/fped.2025.1654973)

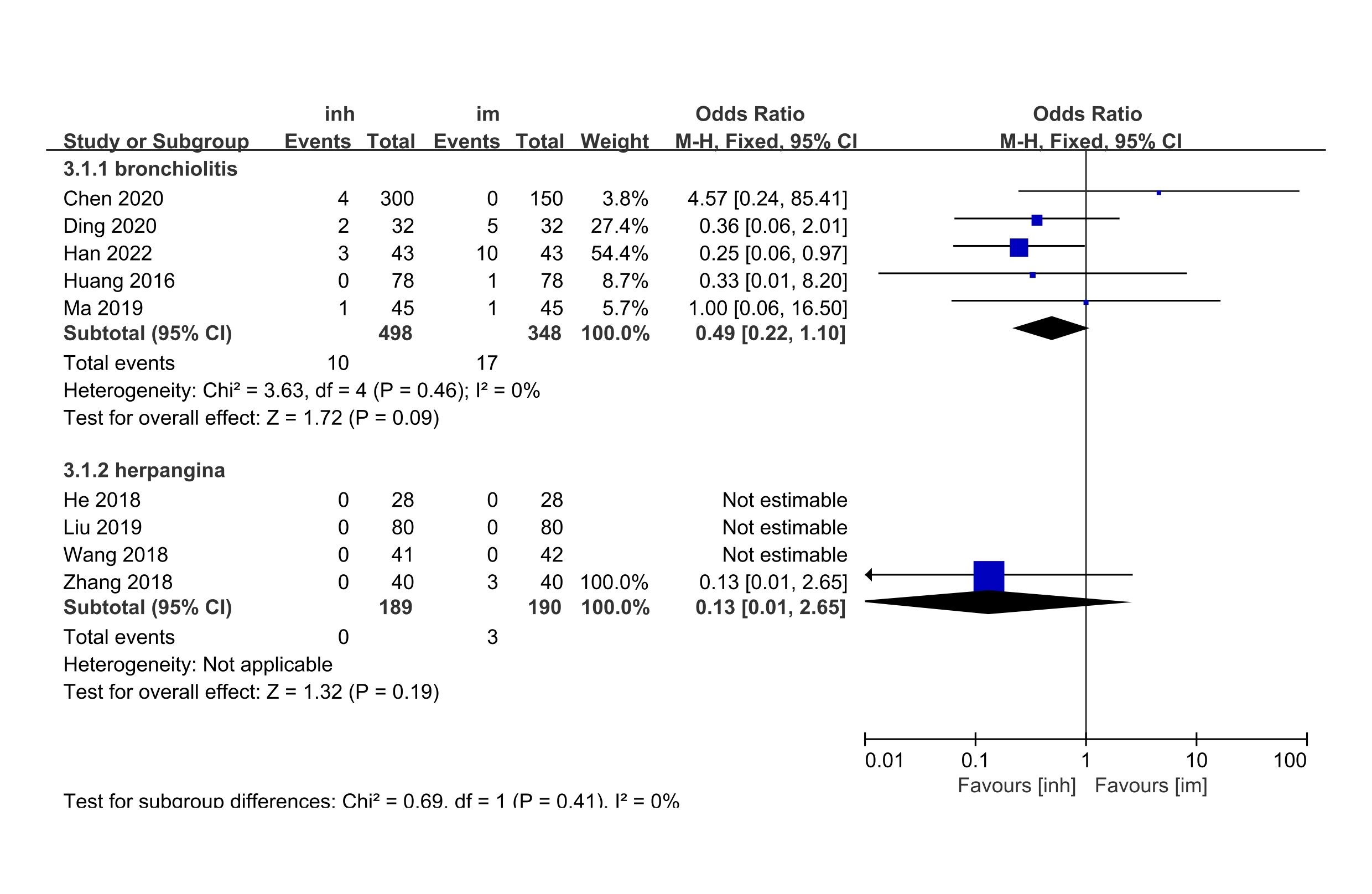

Supplement: Supplementary Figure S1 — Subgroup analysis of adverse reaction incidence rate in inh and im groups. [file Image1.jpeg]
